# Targeting BET Proteins With a PROTAC Molecule Elicits Potent Anticancer Activity in HCC Cells

**DOI:** 10.3389/fonc.2019.01471

**Published:** 2020-01-14

**Authors:** Huapeng Zhang, Gongquan Li, Yi Zhang, Jihua Shi, Bing Yan, Hongwei Tang, Sanyang Chen, Jiakai Zhang, Peihao Wen, Zhihui Wang, Chun Pang, Jie Li, Wenzhi Guo, Shuijun Zhang

**Affiliations:** ^1^Department of Hepatobiliary and Pancreatic Surgery, The First Affiliated Hospital of Zhengzhou University, Zhengzhou, China; ^2^Open and Key Laboratory of Hepatobiliary & Pancreatic Surgery and Digestive Organ Transplantation at Henan Universities, The First Affiliated Hospital of Zhengzhou University, Zhengzhou, China; ^3^Zhengzhou Key Laboratory of Hepatobiliary & Pancreatic Diseases and Organ Transplantation, Zhengzhou, China; ^4^Henan Key Laboratory of Digestive Organ Transplantation, Zhengzhou, China; ^5^Department of Orthopaedic Surgery, The First Affiliated Hospital of Zhengzhou University, Zhengzhou, China

**Keywords:** BET, PROTAC, protein degradation, HCC, apoptosis

## Abstract

**Background and Aim:** Bromodomain and extraterminal domain (BET) family proteins are epigenetic regulators involved in human malignances. Targeting BET proteins for degradation using proteolysis-targeting chimera (PROTAC) recently has drawn increasing attention in the field of cancer therapeutics. BET proteins have been found to be overexpressed in HCC cells and tumor tissues. However, the biological activity of BET-PROTACs in hepatocellular carcinoma (HCC) remains unclear. In this study, we investigated anti-HCC activity of BETd-260, a BET-PROTAC molecule using *in vitro* and *in vivo* models.

**Methods:** BETd-260-mediated anti-HCC activity was investigated by cell viability, apoptosis assays. Efficacy was examined with a cell lines-derived HCC xenograft model in mice. Anticancer mechanism was investigated by RT-PCR, western blotting and immunohistochemical staining.

**Results:** BETd-260 potently suppressed cell viability and robustly induced apoptosis in HCC cells. BETd-260 reciprocally modulated the expression of several apoptotic genes in HCC cells, i.e., suppressing the expression of anti-apoptotic Mcl-1, Bcl-2, c-Myc, and X-linked inhibitor of apoptosis (XIAP), whereas increasing the expression of pro-apoptotic Bad. BETd-260 treatment led to disruption of mitochondrial membrane integrity, and triggered apoptosis via intrinsic signaling in HCC cells. BETd-260 triggered apoptosis in HCC xenograft tissue and profoundly inhibited the growth of HCC xenograft tumors in mice.

**Conclusion:** Our data suggest that pharmacological targeting of BET for degradation may be a novel therapeutic strategy for the treatment of HCC.

## Introduction

Hepatocellular carcinoma (HCC) is one of the most dangerous types of cancer globally. Surgical resection, liver transplantation, trans-arterial chemoembolization and radiofrequency ablation have significantly improved the treatment of HCC in last three decades. However, these treatments offer a survival benefit for only a small fraction of patients with a localized disease. Conventional drugs and targeted agent tyrosine kinase inhibitor sorafeni also are being used in the treatment of HCC. Unfortunately, these systemic therapeutic agents frequently confront drug resistance and severe side effects, which seriously limited the efficacy (1, 2). Therefore, it is imperative to explore novel therapies for this dismal disease.

Bromodomain and extraterminal domain (BET) proteins are epigenetic readers that regulate gene expression and are associated with the formation and progress of many types of cancers (3, 4). Targeting the abnormal expression of BET proteins in cancer cells has been the subject of intensive study in cancer research in the last decade, which has led to the successful development of multiple anticancer agents, such as small molecule inhibitor JQ1 and I-BET151 (5, 6). In a previous study, we have found that bromodomain containing 4 (BRD4), the primary BET family member is overexpressed in most HCC tumor tissues (7). We also have found that JQ1 have anti-proliferative activity in HCC cells, and partially inhibits HCC growth in mice. These data suggest that BET proteins may be rational therapeutic targets for the treatment of HCC.

PROTACs are bifunctional molecules with one side bound to targeted proteins and the other side recognized by the Cullin-dependent E3 ligase. By exploiting the ubiquitin-proteasome system (UPS), PROTAC molecules selectively induce degradation of oncogenic proteins (8, 9). Based on this theory, a few groups recently reported the synthesis of pharmacological BET-PROTAC molecules (10–13). Of note, preclinical studies demonstrated that BET-PROTACs are able to completely eradicate BET proteins from cancer cells, and display remarkable activity against leukemia, prostate, breast cancers, and a few other types of cancer (10–16). However, the anticancer activity of BET-PROTACs in HCC remains uninvestigated. BETd-260 is a newly synthesized BET-PROTAC designed based on a BET inhibitor HJB-97 (10). Herein, we investigated the anti-HCC activity of BETd-260 using *in vitro* and *in vivo* models. Our results showed that compared with small molecule inhibitors, BET degrader BETd-260 displays much stronger anti-HCC activity.

## Materials and Methods

### Cell Lines and Agents

Human HCC BEL-7402, HepG2, SK-HEP-1, SMMC7721, HuH-7, and MHCC97H cell lines were purchased from the China Center for Type Culture Collection (Wuhan, China) and maintained in RPMI1640 or DMEM (HyClone/Thermo Fisher Scientific, Beijing, China) supplemented with 10% heat-inactivated fetal bovine serum (Hangzhou Sijiqing Biological Engineering Materials Co., Ltd., Hangzhou, China). Each cell line was maintained in culture for a maximum of 8 weeks after thawing from frozen. There is no mycoplasma contamination in all 6 HCC cell lines (#05200709001, mycoplasma detection kit, Roche, Shanghai, China).

BETd-260 and HJB-97 were kindly provided by Dr. Shaomeng Wang (University of Michigan at Ann Arbor, MI). JQ1 was obtained from Selleck Chemicals Shanghai (Shanghai, China). All compounds were dissolved in dimethyl sulfoxide (DMSO) at a stock concentration of 10 mmol/L and were stored at −20°C.

### CCK-8 Cell Proliferation Assay

Cell proliferation was determined by Cell Counting Kit 8 (CCK8) (Sigma-Aldrich Shanghai, Shanghai, China). HCC cells were seeded in 96-well culture plates (Costar, Cambridge, MA, USA) at a density of 4,000 cells/well in 100 μL of medium, and treated with serial dilutions of compounds to the desired concentrations in triplicate for 72 h. Before the end of assay, 10 μL CCK-8 was added. After 1–4 h incubation, the absorbance was measured to obtain optical density (OD) values at 450 nm using a microplate reader. OD values for treated cells relative to those of untreated control samples were plotted as a function of drug concentration. Inhibition of cell viability was calculated by the percentage of viable cells relative to the control: % inhibition = 100% × ODT/ODC, where ODT is the average OD value of the treated samples and ODC is the average OD value of the control samples.

### Apoptosis and Cell Death Assays

An apoptosis assay was performed by staining the cells with Annexin-V-FITC/ Propidium iodide (PI) and analyzing apoptosis by flow cytometry with a BD LSR II system (BD Biosciences, Shanghai, China). The assays were performed in duplicate with at least three replications per treatment. Cell death was examined by trypan blue exclusion assays. Cells were stained with trypan blue and dead cells (blue staining) were quantified under a microscopy.

### Western Blotting

Western blot analysis was performed as we described previously (7). The antibodies of BRD2 (A302-583A), BRD3 (A700-069), and BRD4 (A700-005) were purchased from Bethyl Laboratories (Shanghai, China). X-linked inhibitor of apoptosis (XIAP) (#2042), c-Myc Antibody (#9402), PARP (#9542), Caspase-3 (#9662), Bad (#9239), cytochrome c (#11940), and COX IV (#4850) were purchased from Cellular Signaling Technology (Shanghai, China). Mcl-1 (S-19) (#sc-819), Bcl-2 (C-2) (#sc-7382), and Actin (2Q1055) (#sc-58673) were purchased from Santa Cruz Biotechnology (Shanghai, China).

### Subcellular Fractionation

Subcellular fractionation was performed as we described previously (7). Briefly, cells were homogenized using an ice-cold cylinder cell homogenizer (20–25 strokes). Homogenized cell lysates were separated by centrifugation at 750 g for 10 min, and the supernatants were further centrifuged at 10,000 g for 20 min. The remaining supernatant was used as the cytosolic fraction and subjected to western blot analysis.

### qRT-PCR Assay

For qRT-PCR, total RNA was isolated using TRIzol reagent (Invitrogen), and cDNA was synthesized using the high capacity cDNA archive kit (Applied Biosystems). The mRNA level of Bad was quantified by qRT-PCR using SYBR Premix Ex Taq (Applied Takara Bio, Shanghai, China). The Bcl-2-associated death promoter (Bad) primers were as follows: (forward) 5′- CCCAGAGTTTGAGCCGAGTG-3′ (reverse), 5-CCCATCCCTTCGTCGTCCT-3. The gapdh primers were as follows: (forward) 5-TGCCTCCTGCACCACCAACT-3, and (reverse) 5- CGCCTGCTTCACCACCTTC-3. The PCR conditions included an initial denaturation step of 95°C for 2 min, followed by 35 cycles of 95°C for 10 s, 56°C for 20 s, and 72°C for 20 s, and a final elongation step of 72°C for 10 min. Quantitation relative to the endogenous control (GAPDH) was performed using the Applied Biosystems 7500 Fast System SDS software.

### RNA Interference

siRNAs oligos against Bad, caspase-9 and caspase-8 were obtained from GE Dharmacon (Shanghai, China). The siRNA transfections (100 pmol/L) were performed using Lipofectamine RNAiMax transfection reagent (Invitrogen, Shanghai, China).

### Immunohistochemistry (IHC)

IHC was performed as we described previously (7). Xenograft tumor tissues were obtained from Balb/c tumor-bearing mice treated with single dose of 5 mg/kg BETd-260 or vehicle control. The following antibodies were used for IHC: BRD2 (IHC-00612), BRD4 (HC-00396), BAD (A302-384A) from Bethyl Laboratories (Shanghai, China); BRD3 (ab264420) from Abcam (Shanghai, China); cleaved PARP (Asp214) (#32563), activated caspase-3 (#9664), and Ki-67 (8D5) (9449) from Cell Signaling Technology (CST, Shanghai, China), Anti-Mcl-1 (MAB828) from R&D Systems (R&D Systems, Shanghai, China).

### Animal Studies

All *in vivo* studies were conducted under an animal protocol approved by Zhengzhou University Committee on Use and Care of Animals. All animals received humane care according to the criteria outlined in the “Guide for the Care and Use of Laboratory Animals Chinese Version” (2006). HCC cells (5 million cells per tumor) suspended in 0.1 mL of Matrigel were injected subcutaneously into the flanks of 6-week-old Balb/c mice (Charles River, Beijing, China) to establish subcutaneous tumors. For efficacy studies, when tumor grafts had reached an average volume of 100 mm^3^, mice were randomized into treated or control groups, with eight mice in each group. BETd-260 (5 mg/kg) or vehicle control (10% PEG400: 3% Cremophor: 87% PBS, 2% TPGS: 98% PEG200) was dosed intravenously (iv) as indicated. Tumor volume and animal weights were monitored 1–3 times every week. Tumor volume was measured from two directions with a digital caliber and calculated as follows: Tumor volume (mm^3^) = (length × width^2^)/2. Tumor growth inhibition (TGI) was calculated according to the formula [1 – (T – T0)/(C – C0)] × 100, where T and T0 are the mean tumor volumes at Day 22 and Day 1, respectively, for the experimental group, C and C0 are mean tumor volumes for the vehicle control group. For the pharmacodynamics study, when tumors reached the size of 200 mm^3^, mice treated with a single dose of BETd-260 or vehicle control were euthanized at 24 h time points, and tumor tissues were harvested from animals and post-fixed in 4% paraformaldehyde and or in liquid nitrogen.

### Statistical Analysis

The results were displayed as the mean ± standard error of the mean (SEM) unless otherwise specified, and were compared with an Unpaired *t*-test using GraphPad Prism 7 software. *p* < 0.05 was deemed significant.

## Results

### BETd-260 Induces BRD2, BRD3, and BRD4 Degradation in a Panel of HCC Cell Lines

**Figure 1:**
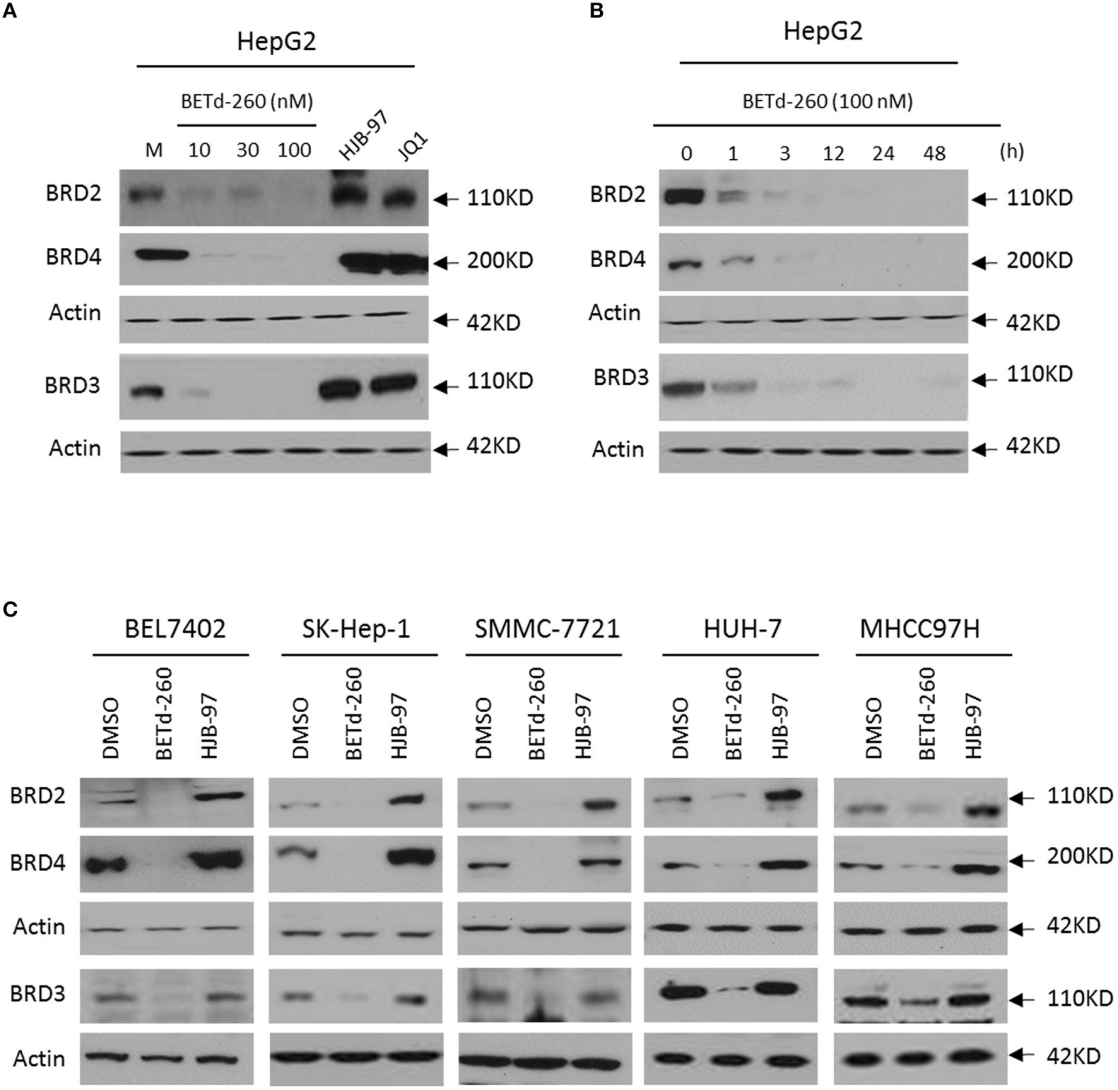
BETd-260 induces degradation of BRD2, BRD3, and BRD4 in HCC cells. **(A)** HepG2 cell line was treated by BETd-260, HJB-97, or JQ1 as indicated for 24 h. The protein levels of BRD2, BRD3 and BRD4 were examined by western blot analysis. Actin was used as a loading control. **(B)** HepG2 cell line was treated by BETd-260 at 100 nmol/L for different times. The protein levels of BRD2, BRD3, and BRD4 were examined by western blot analysis. Actin was used as a loading control. **(C)** BEL-7402, SK-HEP-1, SMMC-7721, HuH-7, and MHCC97H cell lines were treated by BETd-260 at 100 nmol/L for 24 h. The protein levels of BRD2, BRD3 and BRD4 were examined by western blot analysis. Actin was used as a loading control. Data are representative of three independent experiments.

We initiated our study by examining the effect of BETd-260 on the protein levels of BRD2, BRD3, and BRD4 in HepG2 cell line by western blotting. The results showed that treatment with BETd-260 at a dose-range of 10–100 nmol/L for 24 h almost completely deleted three oncogenic BET proteins from the cells (Figure 1A). A time course study showed that treatment with BETd-260 for 1 h largely reduced the level of BRD2, BRD3, and BRD4 proteins and for 12 h completely eliminated the BET proteins in the HCC cells (Figure 1B). These results suggest that the degradation activity of BETd-260 is rapid and robust in this cell line. We further treated 5 more HCC cell lines that included BEL-7402, SK-HEP-1, SMMC-7721, HuH-7, and MHCC97H with BETd-260 at 100 nmol/L for 24 h and examined the effect on the BET proteins. The results showed that BETd-260 completely degraded the BET proteins in BEL-7402, SK-HEP-1, SMMC-7721 cell lines and reduced BET proteins to very low levels in HuH-7 and MHCC97H cell lines (Figure 1C). These results demonstrate that BETd-260 has broad BET-degradation activity in HCC cells. In contrast, BET inhibitors HJB-97 at 1,000 nmol/L and JQ1 at 1,000 nmol/L had no inhibitory effect on the levels of three BET proteins in these HCC cell lines.

### BETd-260 Potently Suppresses Viability of HCC Cells in a Dose-Dependent Manner

**Figure 2:**
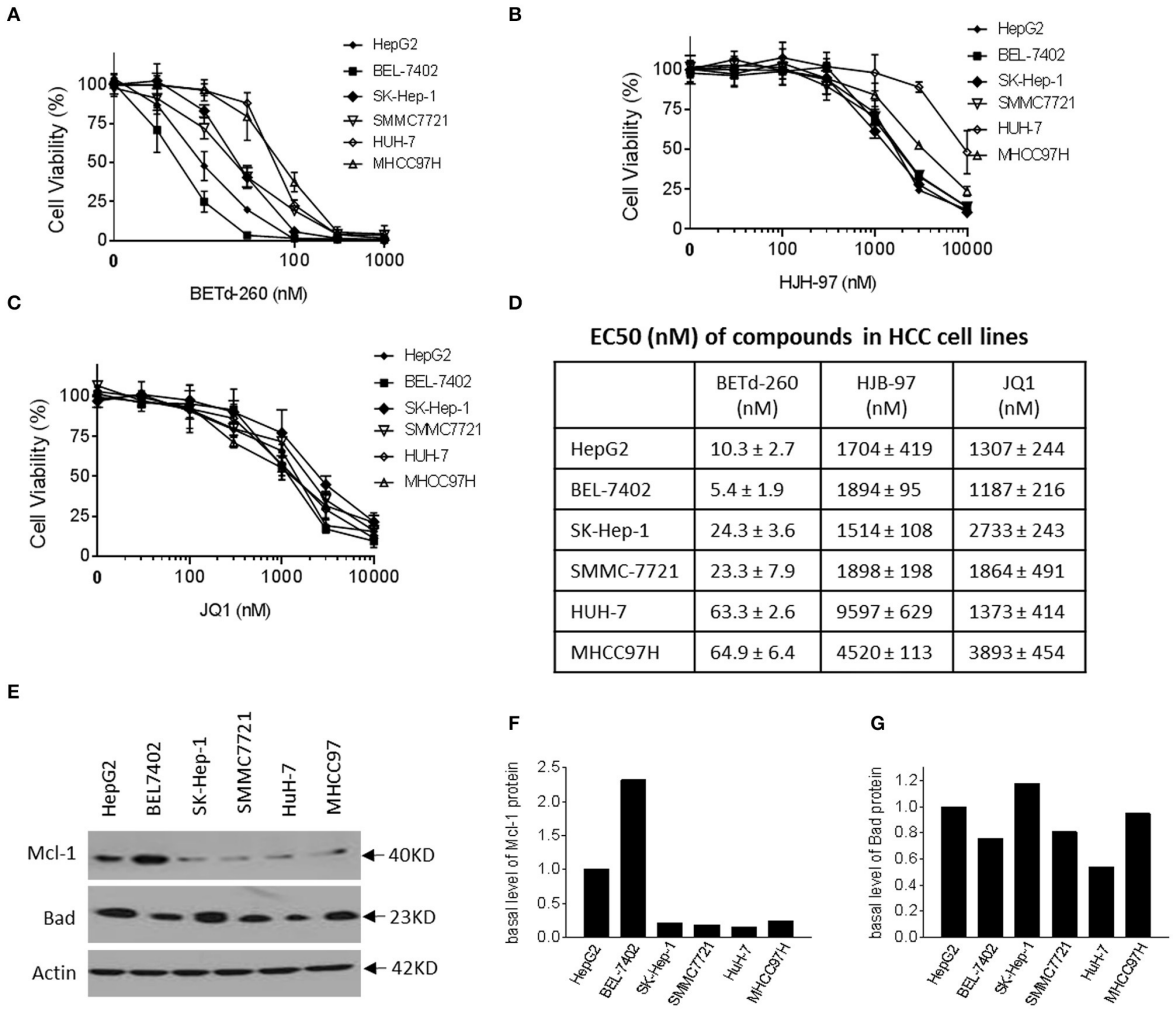
BETd-260 potently inhibits cell viability in HCC cells. HepG2, BEL-7402, SK-HEP-1, SMMC-7721, HuH-7, and MHCC97H cell lines were treated with serially dilutions of **(A)** BETd-260, **(B)** HJB-97, or **(C)** JQ1 as indicated for 72 h. Cell viability was examined by CCK-8 assay. **(D)** The EC50 values were calculated with Prism 7 software and listed in the table. Data are mean ± SEM (*n* = 4). **(E–G)** HCC cells growing in exponential stage were harvested. **(E)** The expression of Mcl-1 and Bad were examined by western blot analysis. Actin was used as a loading control. **(F, G)** The levels of Mcl-1 and Bad proteins were quantified with ImageJ software, and the values were plotted. Data are representative of two independent experiments.

We next examined the effect of BETd-260 and BET inhibitors HJB-97 and JQ1 on cell viability in the abovementioned 6 HCC cell lines. The results of CCK-8 assay showed that the degrader dose-dependently inhibited cell viability in the 6 HCC cell lines in Figure 2A. These results suggest that all these HCC cell lines are sensitive to the BETd-260. In contrast, the BET inhibitors had much weaker activity (Figures 2B,C). Specifically, BETd-260 achieved low nanomolar EC_50_ values in all 6 HCC cell lines, while HJB-97 achieved 1,514–9,597 nmol/L EC_50_ values and JQ1 had EC_50_ values 1,187–3,893 nmol/L EC_50_ values, respectively, in the 6 HCC cell lines Figure 2D).

Of note, the results also indicated that BETd-260 at nanomolar concentrations eliminated all cell viability in the 6 HCC cell lines, suggesting a cytotoxic activity (Figure 2A). In contrast, the BET inhibitors could not completely suppress the cell viability even at as high as 10,000 nmol/L (Figures 2B,C).

We examined the basal levels of Mcl-1 and Bad in the HCC cell lines by western blotting (Figures 2E–G). The results showed that all 6 HCC cell lines expressed appreciable levels of both Bcl-2 family members. Of note, HepG2 and BEL-7402, the two most sensitive cell lines expressed a distinct higher level of Mcl-1, a critical survival protein (Figures 2E–G).

### BETd-260 Triggers Massive Apoptosis Through Intrinsic Signaling Pathway in HCC Cells

**Figure 3:**
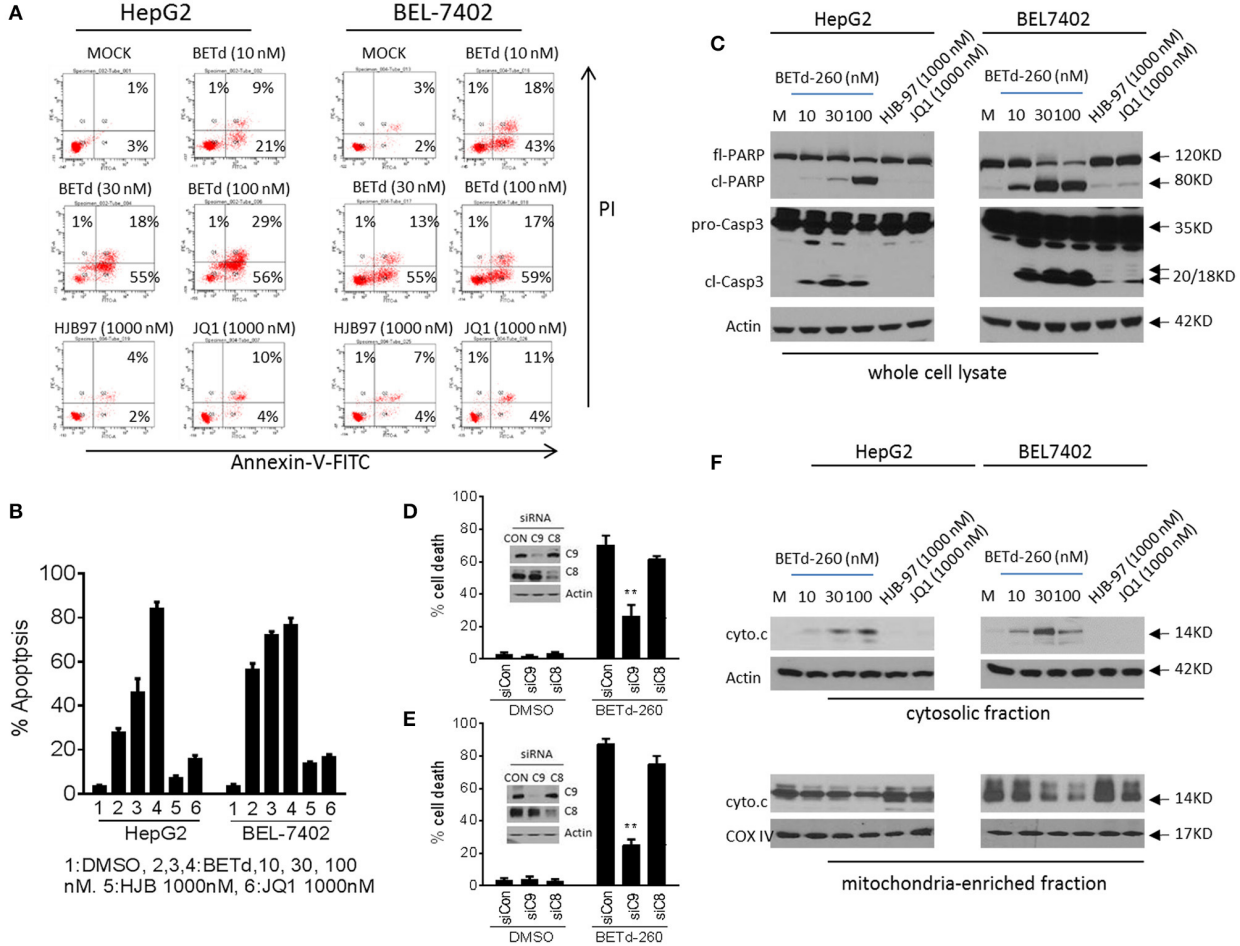
BETd-260 triggers massive apoptosis in HCC cells. **(A)** HepG2 and BEL-7402 cell lines were treated with BETd-260, HJB-97 or JQ1 as indicated for 48 h. Apoptosis was examined by propidium iodide (PI)/Annexin-V staining in combination with flow cytometry assay. **(B)** Average percentages of apoptosis from three independent experiments were plotted in the graphs. **(C)** HepG2 and BEL-7402 cell lines were treated for 24 h. Treated cells were lysed and the protein levels of full-length PARP (fl-PARP), cleaved PARP (cl-PARP), pro-Caspase3 (pro-Casp3), and cleaved caspase-3 (cl-Casp3) were examined by western blot analysis. Actin was used as a loading control. **(D)** HepG2 and **(E)** BEL-7402 cell lines were transfected with siRNA against caspases-9,−8, for 24 h. The transfected effectiveness was examined by western blotting (inserts). The transfected cells were treated by BETd-260 at 100 nmol/L for another 48 h. The cell death was determined by trypan blue assay and cell death percentages were plotted. **(F)** HepG2 and BEL-7402 cell lines were treated for 24 h. Treated cells were cell fractionated into cytosolic and mitochondria-enriched membranes fractions, and the protein level of cytochrome c was examined by western blot analysis. Actin was used as a loading control for cytosolic fraction and COX IV was used as a loading control for mitochondrial enriched membranes fraction. Data are representative of three independent experiments. **p < 0.01.

To investigate whether the cytotoxic activity of BETd-260 could be attributed to apoptosis induction, we treated HepG2 and BEL-7402 cell lines with BETd-260, HJB-97 or JQ1 for 48 h, and examined apoptosis with FACS analysis of Annexin V/PI staining. The results showed that BETd-260 at 10 nmol/L effectively triggered apoptosis in the both HCC cell lines and at 100 nmol/L induced 86 and 77% cells apoptosis, respectively, in HepG2 and BEL-7402 cell lines (Figures 3A,B). The same treatment also induced massive (45–85%) cell death in other four HCC cell lines (SI Figure 1A). These observations demonstrated that BETd-260 had strong apoptotic effect in HCC cells. Western blotting was performed to examine the activation of apoptosis signaling and the results showed that treatment with BETd-260 for 24 h resulted in extensive cleavage of PARP and robust activation of caspase-3 in all 6 HCC cell lines (Figure 3C and SI Figure 1B). The results suggest that apoptosis is deeply involved in BETd-260-mediated anti-HCC activity.

We next silenced two initiator caspases with siRNA to investigate which pathway is more important in BETd-260-induced anti-HCC activity. The data showed that knockdown of caspase-9, the initiator caspase of intrinsic pathway, but not knockdown of caspase-8, the initiator caspase of extrinsic pathway significantly attenuated BETd-260-triggered cell death (Figures 3D,E). These observations suggest that intrinsic pathway plays an essential role, whereas caspase-8-initiated extrinsic pathway is dispensable in the cytotoxic effect of the degrader. Because intrinsic apoptosis signaling is mediated by mitochondria (17), we thus further conducted subcellular fractionation assay to examine the effect of BETd-260 on mitochondrial membrane integrity. The results showed that BETd-260 treatment led to an increase of cytochrome c in cytosolic fraction, accompanied by a decrease of cytochrome c in mitochondria-enriched membrane fraction in the two HCC cell lines (Figure 3F), suggesting cytosolic release of cytochrome c. These results further substantiate that BETd-260 triggers apoptosis via intrinsic signaling pathway in HCC cells.

In contrast, the BET inhibitors at 1,000 nmol/L had only negligible apoptotic activity in these two HCC cell lines (Figures 3A–F).

### BETd-260 Reciprocally Modulates the Transcription of Apoptosis Related Genes, and Represses the Expression of c-Myc in HCC Cells

In order to explore the potential anti-HCC mechanism of BETd-260, we analyzed the effect of BETd-260 on expression of several known downstream targets of BET proteins, including Bcl-2, Mcl-1 and c-Myc, as well as XIAP (5, 6, 14).

**Figure 4:**
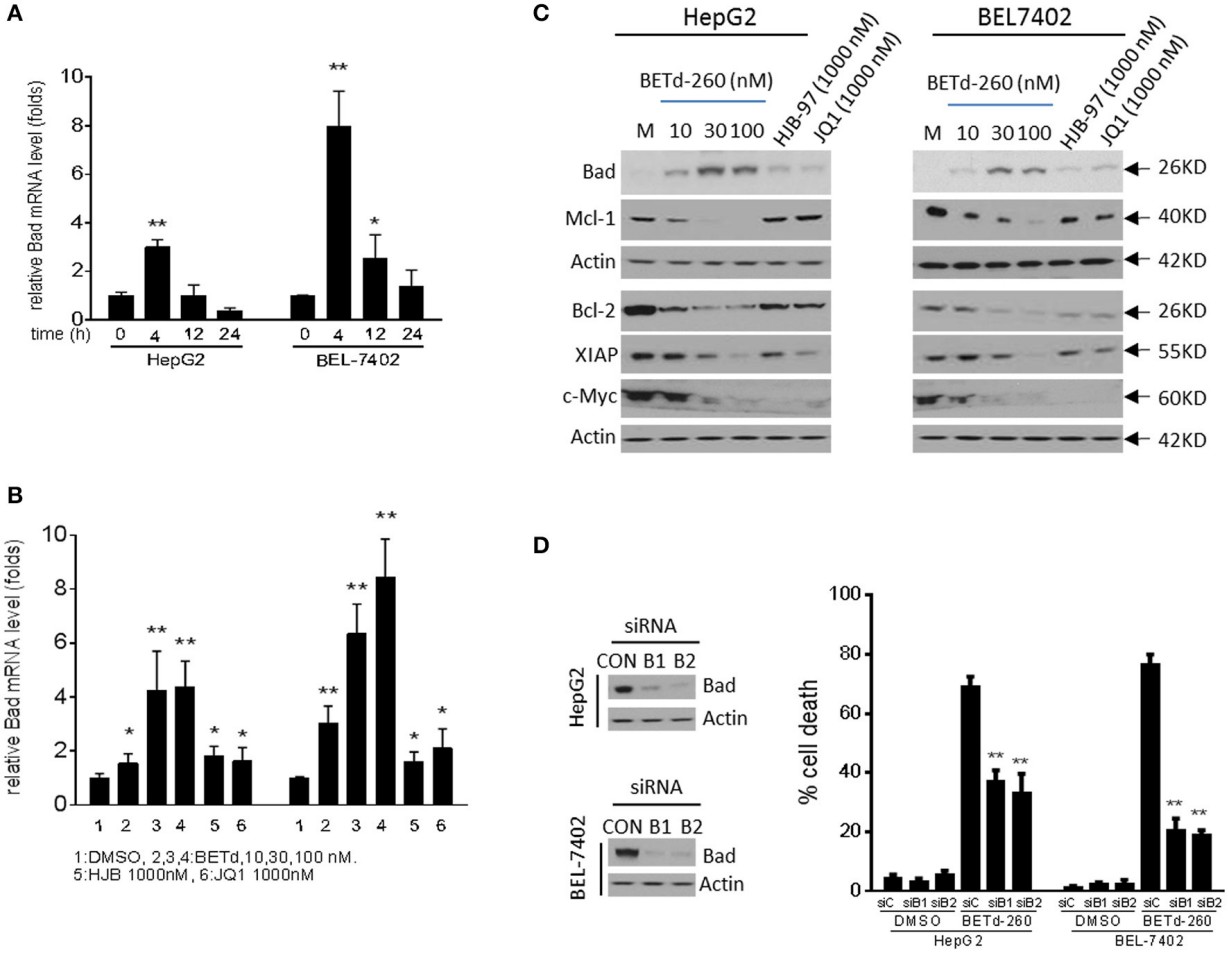
BETd-260 reciprocally modulates the transcription of apoptosis related genes, and inhibits the expression of c-Myc in HCC cells. **(A)** HepG2 and Bel-7402 cell lines were treated by BETd-260 at 100 nmol/L for 4, 12, and 24 h. Bad mRNA expression was analyzed using qRT-PCR. **(B)** HepG2 and Bel-7402 cell lines were treated by BETd-260, HJB-97, or JQ1 as indicated for 4 h. Bad mRNA expression was analyzed using qRT-PCR. **(C)** HepG2 and Bel-7402 cell lines were treated by BETd-260, HJB-97 (1,000 nM), or JQ1 (1,000 nM) as indicated for 24 h. The expressions of Bad, Mcl-1, Bcl2, XIAP, and c-Myc were examined by western blotting analysis of whole cell lysates. Actin was used as a loading control. **(D)** HepG2 and BEL-7402 cell lines was transfected with control siRNA or Bad siRNA (B1 and B2) for 24 h (left panels). Transfected effectiveness was examined by western blot (right panel). Transfected cells were treated by BETd-260 at 100 nmol/L for 48 h. Cell death was examined with trypan blue exclusion assays. Data are representative of three independent experiments. *p < 0.05; **p < 0.01.

RT-PCR results showed that BETd-260 inhibited the transcription of Mcl-1 and up-regulated the expression of Bad (Figures 4A,B and SI Figure 2). Western blotting showed that treatment by the degrader led to reduction of Mcl-1, Bcl-2, and XIAP, and increase of Bad at protein level in HepG2 and BEL-7402 cell lines (Figure 4C). These results suggest that the BET degrader actively regulates the expression of several apoptosis related proteins in HCC cells.

As Bad is a potent pro-apoptotic Bcl-2 protein that binds and antagonizes multiple anti-apoptotic Bcl-2 proteins, including Bcl-xl, Bcl-w, and Bcl-2 (17, 18), we reasoned that Bad was involved in BETd-260-triggered apoptotic cell death. To prove this, we silenced Bad to examine the inhibitory effect on cell death induction in HepG2 and BEL-7402 cells. The results showed that knockdown of Bad by siRNA transfection significantly attenuated BETd-260-induced cell death in HepG2 and BEL-7402 cell lines (Figure 4D). These results suggest that Bad plays an essential role in BETd-260-mediated anti-HCC activity.

Previous studies have shown that BET inhibitors and BET degraders potently suppressed oncogenic protein c-Myc in multiple types of cancers (5, 14). We next investigated the effect of BETd-260 on c-Myc in HCC cells. The results of western blot showed that BETd-260 treatment distinctly reduced the level of c-Myc protein in 5 out of 6 HCC cell lines (Figure 4C and SI Figure 3). These results suggest that BET degrader also potently target c-Myc in most HCC cells.

### BETd-260 Induces BET Degradation, Modulates the Expression of Mcl-1, and Bad, Triggers Apoptosis *in vivo*

**Figure 5:**
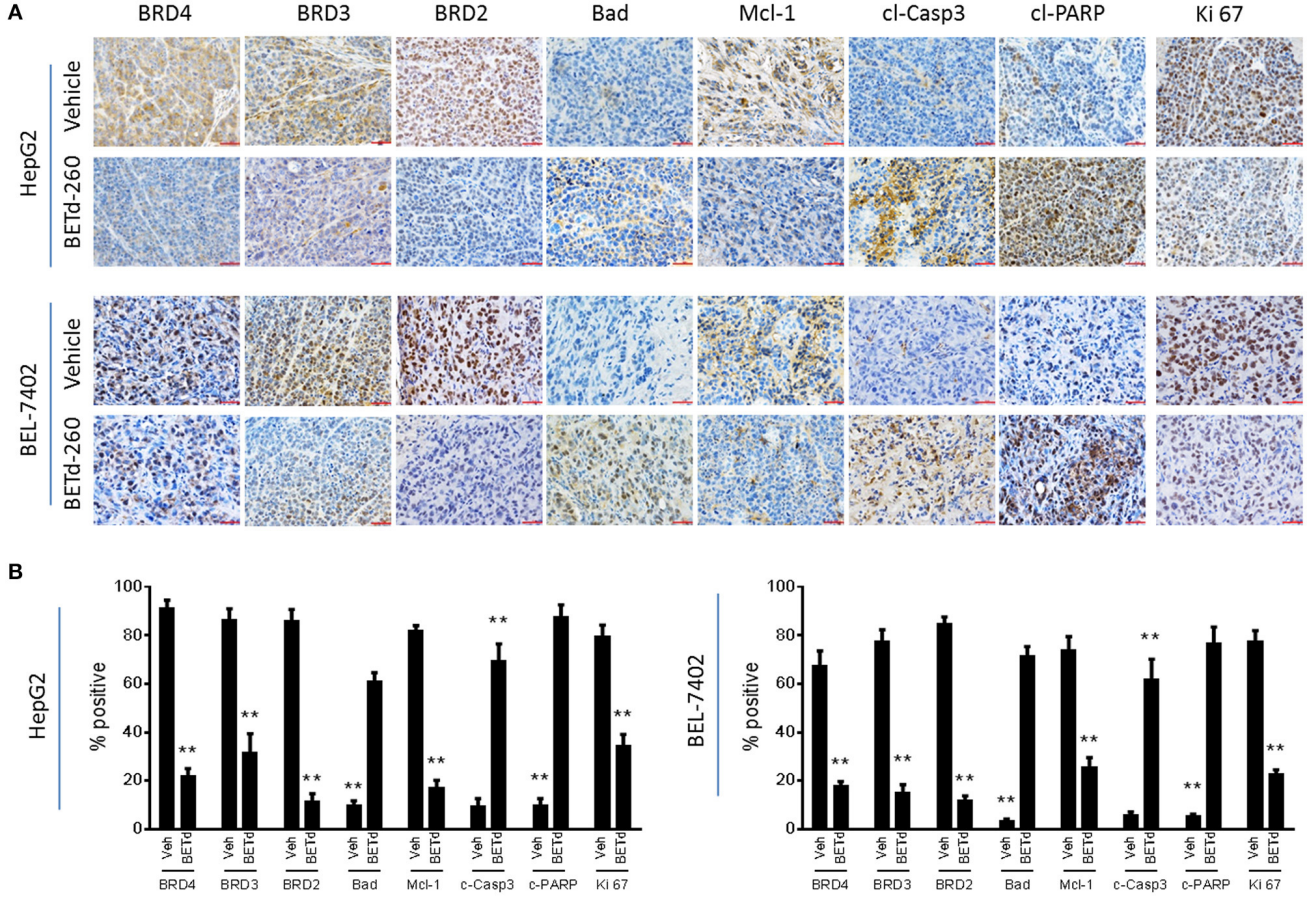
BETd-260 induces BET degradation, triggers apoptosis, and inhibits proliferation in HCC xenograft tissue in mice. BALB/c mice bearing HepG2 and BEL-7402 xenograft tumors were treated by a single intravenous dose of 5 mg/kg BETd-260 (BETd) for 24 h or vehicle (Veh). **(A)** Two to three mice were sacrificed and tumor tissue was harvested. The expression of BRD2, BRD3, and BRD4, Mcl-1, Bad, activated caspase-3, cleaved PARP, and Ki 67 was examined by immunohistochemistry staining. Representative photographs were presented. **(B)** The percentages of HCC tumor cells positively stained with BRD2, BRD3, and BRD4, Mcl-1, Bad, cleaved caspase-3 (c-Casp3), cleaved PARP (c-PARP), and Ki 67 were quantified under microscopy, and plotted. Data are representative of three independent experiments. **p < 0.01.

We next investigated the *in vivo* biological activity of BETd-260 using HCC xenograft models in mice. Mice bearing HCC xenograft tumors were treated with BETd-260 and the xenograft HCC tumor tissues were harvested and analyzed by IHC. The results showed that treatment with a single dose (5 mg/kg) of BETd-260 for 24 h significantly suppressed the expression of BRD2, BRD3 and BRD4 in tumor tissue of both HCC models (Figures 5A,B). IHC assays also showed that BETd-260 treatment significantly reduced the expression of Mcl-1 and distinctly increased the expression of Bad in HCC xenograft tissue. Moreover, BETd-260 treatment augmented the expression of two typical apoptosis biomarkers, cleaved form of PARP, activated form of caspase-3 in HCC tumor tissue, suggesting that BET-PROTAC BETd-260 reciprocally modulates Mcl-1 and Bad, triggers apoptosis in HCC xenograft tumor tissues. Additionally, the IHC analysis also showed that BETd-260 treatment reduced the expression of Ki 67 by 57 and 71%, respectively, in HepG2 and BEL-7402 tumor tissues (Figures 5A,B), suggesting the growth of HCC cells in xenograft tumor tissue was largely inhibited by BETd-260.

### BETd-260 Shows Potent anti-HCC Efficacy *in vivo*

**Figure 6:**
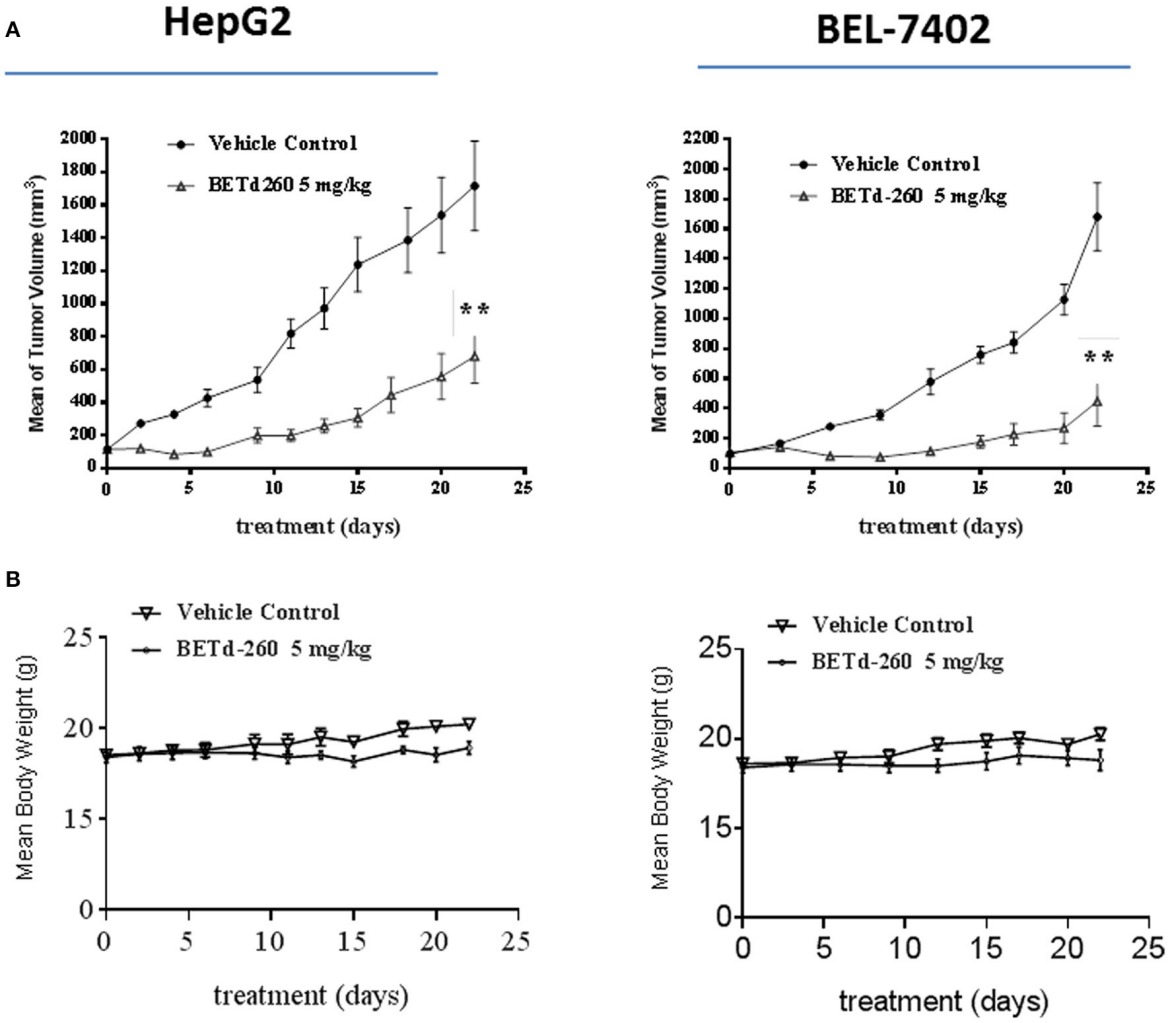
BETd-260 profoundly inhibits tumor growth of HCC xenografts in mice. BALB/c mice bearing HepG2 and BEL-7402 xenograft tumors were treated by BETd-260 and vehicle control intravenously 3 times per week for 3 weeks. Tumor sizes and body weights were measured every 2–3 days. Data are mean ± *SEM* (*n* = 7–8). **(A)** Tumor values were plotted with Prism 6 software. **(B)** Body weights were plotted with Prism 7 software. *p*-values between each treated and the vehicle control group were determined using 2-way ANOVA. **p < 0.01.

We next studied the *in vivo* anti-tumor effect of BETd-260 in mouse xenograft models. HepG2 and BEL-7402 cell lines were used to create subcutaneous HCC xenograft tumors in Balb/c mice. Tumor bearing mice were treated by either vehicle control or BETd-260 at the dose of 5 mg/kg intravenously (iv), 3 times per week for 3 weeks. Compared to the vehicle control, treatment by BETd-260 significantly inhibited tumor growth in both HCC models (two-way anova, *P* < 0.01) (Figure 6A). The average tumor weight in the mice treated with BETd-260 on day 22 after the treatment was significantly lower than those in the mice treated with vehicle in both HepG2 and BEL-7402 models (Figure 6B). BETd-260 achieved TGI 49 and 78%, respectively, in HepG2 and BEL-7402 models. These results suggested that BETd-260 have profound anti-HCC efficacy. Additionally, BETd-260 treatment only slightly affected body weight of mice in both models, suggesting no or weak toxicity during the treatment.

## Discussion

HCC is one of the most lethal cancers. Systematic or localized chemotherapy are commonly used treatments for patients with HCC. However, HCC are intrinsically resistance to these treatments due to the deregulations of cancer-related genes. Current evidences indicate that epigenetic alterations are responsible, at least partially for gene deregulations in HCC cells (19, 20). BET family proteins are cancer-related epigenetic regulators. Targeting BET with PROTACs has been reported to have potent anticancer activity against breast, prostate and Merkel cell cancer and leukemia (3–7, 16). In an effort to explore more effective treatment methods for HCC, we here investigated the anti-HCC activity of BET-PROTACs. Our data demonstrate that BETd-260 displays superior anticancer activity against HCC as compared with its corresponding BET inhibitor HJB-97 and the prototype BET inhibitor JQ1. To the best of our knowledge, this is the first study reporting the therapeutic potential of BET-PROTAC in HCC. The findings may provide supporting evidences for testing these novel agents in the management of HCC.

Based on the results of cell-based apoptosis, caspases knockdown and subcellular fractionation assays, we conclude that apoptosis plays a key role in BETd-260-mediated anti-HCC activity. Recent reports showed that BET-PROTACs also provoke robust apoptotic activity in leukemia, and in other solid cancer cells (3–7, 16). Therefore, these evidences strongly suggest that apoptosis is a key mechanism by which BET-PROTACs execute their anti-cancer activity. In contrast, BET inhibitors affect cancer cells largely through cytostatic effect (14, 21).

Intrinsic apoptotic pathway is stringently controlled by the Bcl-2 family member proteins, as well as inhibitor of apoptosis (IAP) protein family members (17, 22). Findings from complementary approaches including RNA-seq RT-PCR and western blotting indicate that BETd-260 very efficiently inhibits the expression of Bcl-2 and Mcl-1 in HCC cells. This is in agreement with the role of BET proteins as a co-activator for the transcription of these prosurvival members (23, 24).

Abnormal expression of Bcl-2, Mcl-1, and Bad has been documented to be the key factors of apoptosis resistance in HCC (25–30). For instance, Sieghart et al. found that Mcl-1 was overexpressed in 51% HCC, irrespective of underlying disease (26). In the meantime, Galmiche et al. and Hu et al. found that the expression of Bad is profoundly suppressed in HCC (28–30). In this study, we investigated the relationship between BETd-260 sensitivity and the expression of Mcl-1 and Bad in HCC cell lines. Our data showed that the two most sensitive HCC cell lines (HepG2 and BEL-7402) express much higher level of Mcl-1. This finding suggests that Mcl-1 represents a key resistant factor in apoptosis induction in these two HCC cell lines. Therefore, our results altogether indicate that by concomitantly targeting these abnormalities in a reciprocal manner, BET-PROTAC could be used as unique agents to overcome apoptosis resistance in HCC cells.

Noteworthily, our data also indicate that the degrader distinctly suppresses the expression of XIAP and c-Myc in HCC cells. Since XIAP directly inhibits apoptosis signaling through suppressing the activity initiator caspase-9 and the effector caspase-3 (22), and c-Myc indirectly inhibits apoptosis signaling pathway through target genes (31–33), these evidences indicate that BETd-260 elicits apoptosis in HCC cells by targeting multiple apoptosis related genes.

IHC analysis revealed that BETd-260 suppresses Mcl-1 expression, increases Bad expression, and triggers robust apoptosis in HCC xenograft tumor tissues. These observations suggest that BETd-260 might act through the same mechanism in HCC xenograft tumor tissues as in HCC cells. The efficacy study showed that BETd-260 remarkably inhibits the growth in both HepG2 and BEL-7402 models. This exceptional activity is in striking contrast to the modest efficacy of JQ1 alone, as reported by our group previously (7). This evidence suggests that complete depletion of BET proteins would have much better efficacy in HCC than BET inhibition, likely attributed to the strong apoptotic cell killing effect by the BET-PROTAC.

Overall, this study demonstrates that by inducing apoptosis, the BET-PROTAC elicits strong anti-HCC activity, suggests that BET-PROTACS may have beneficial effect in the management of HCC. Nonetheless, since BET proteins are associated with the transcription of numerous other genes, further studies should be conducted to decipher the involvement of these genes in BET-PROTACs mediated anti-HCC activity.

## Data Availability Statement

The raw data supporting the conclusions of this manuscript will be made available by the authors, without undue reservation, to any qualified researcher.

## Ethics Statement

All *in vivo* studies were conducted under an animal protocol approved by Zhengzhou University Committee on Use and Care of Animals.

## Author Contributions

HZ, GL, and YZ designed the study, performed the experiments, and drafted the manuscript. JS, BY, HT, and SC carried out the experiments and drafted the manuscript. JZ, PW, ZW, and CP performed the *in vivo* experiment. JL and WG carried out flow cytometry. SZ designed the study and revised the manuscript. All authors read and approved the final manuscript.

### Conflict of Interest

The authors declare that the research was conducted in the absence of any commercial or financial relationships that could be construed as a potential conflict of interest.

## Abbreviations

Bad: BCL-2-Associated Agonist of Cell Death
Bcl-2: B-cell lymphoma-2
BET: Bromodomain and extraterminal domain
BRD2: Bromodomain 2
BRD3: Bromodomain 3
BRD4: Bromodomain 4
ChiP: Chromatin-immunoprecipitation
DMSO: Dimethyl sulfoxide
HCC: Hepatocellular carcinoma
IHC: Immunohistochemistry
Mcl-1: Myeloid Cell Leukemin-1
PARP: Poly ADP-ribose polymerase
PROTAC: Proteolysis-targeting chimera
qRT-PCR: Real-Time Quantitative Reverse Transcription PCR
RNA: Ribonucleic Acid
siRNA: Small Interfering RNA
UPS: Ubiquitin-proteasome system.
