# Supplementary figures and images for: Targeting BET Proteins With a PROTAC Molecule Elicits Potent Anticancer Activity in HCC Cells
Source: Front Oncol. 2020 Jan 14;9:1471. doi: 10.3389/fonc.2019.01471 (PMC6971110; doi:10.3389/fonc.2019.01471)

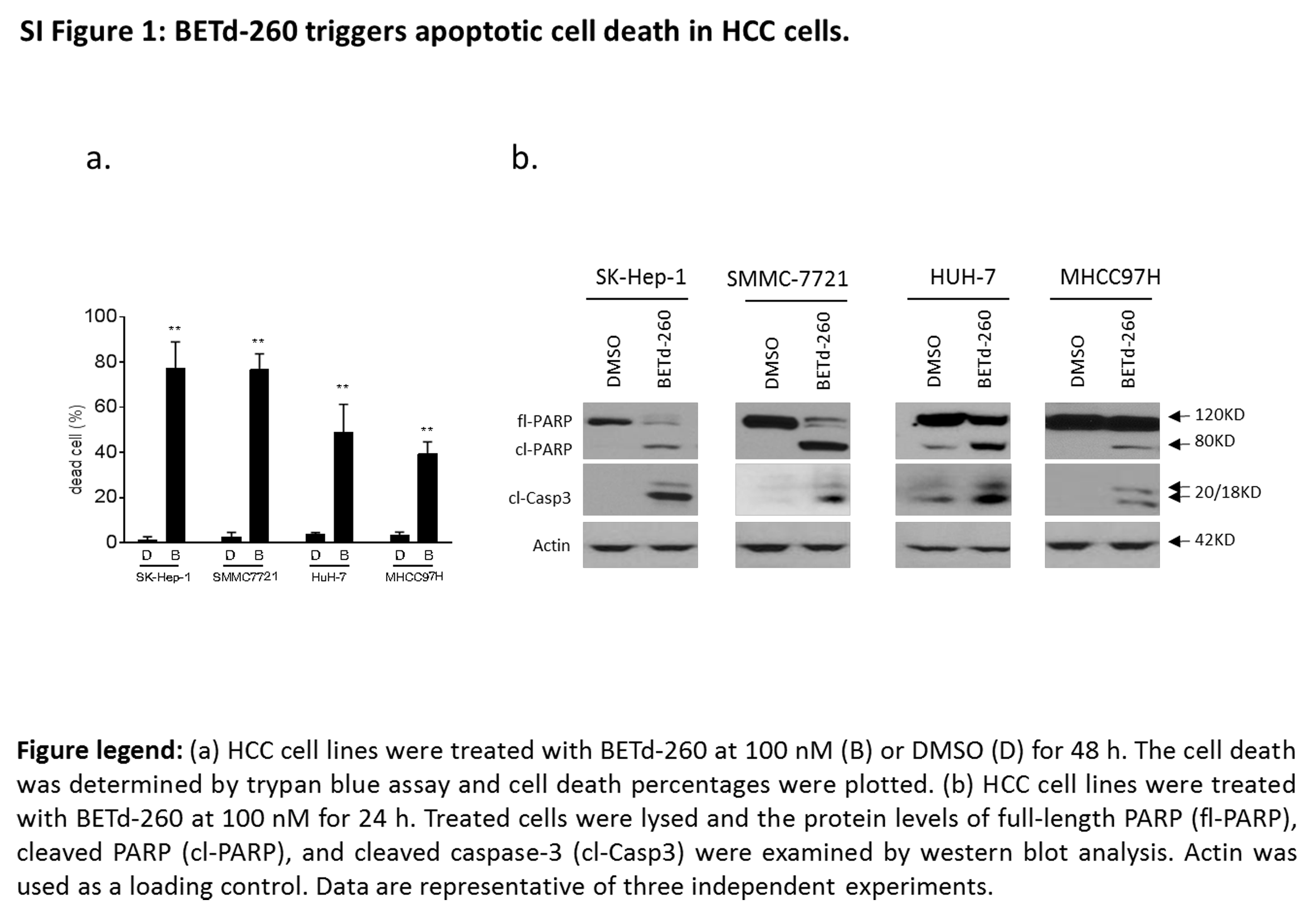

Supplement: Supplementary file 1 [file Image_1.TIF]

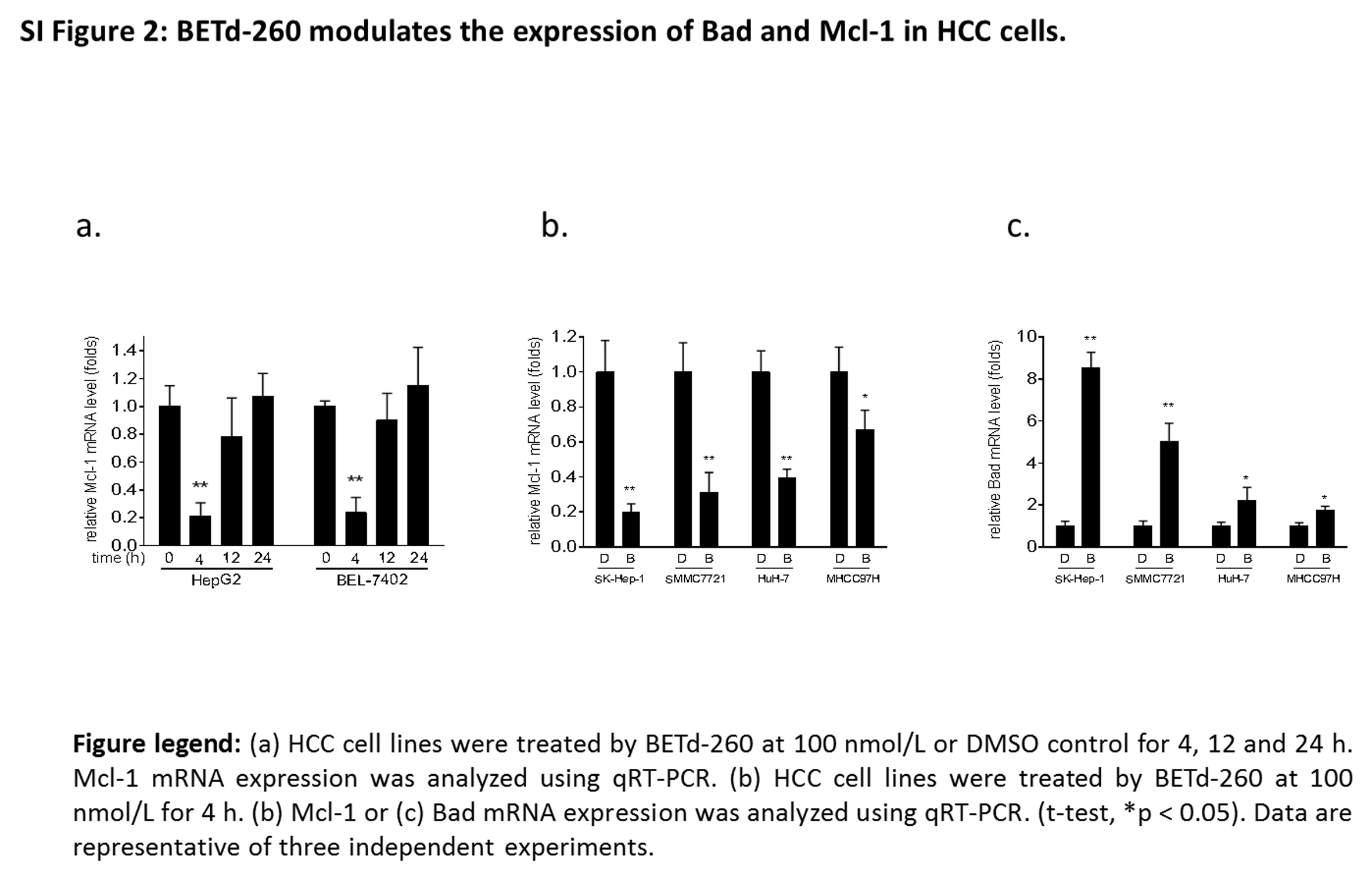

Supplement: Supplementary file 2 [file Image_2.TIF]

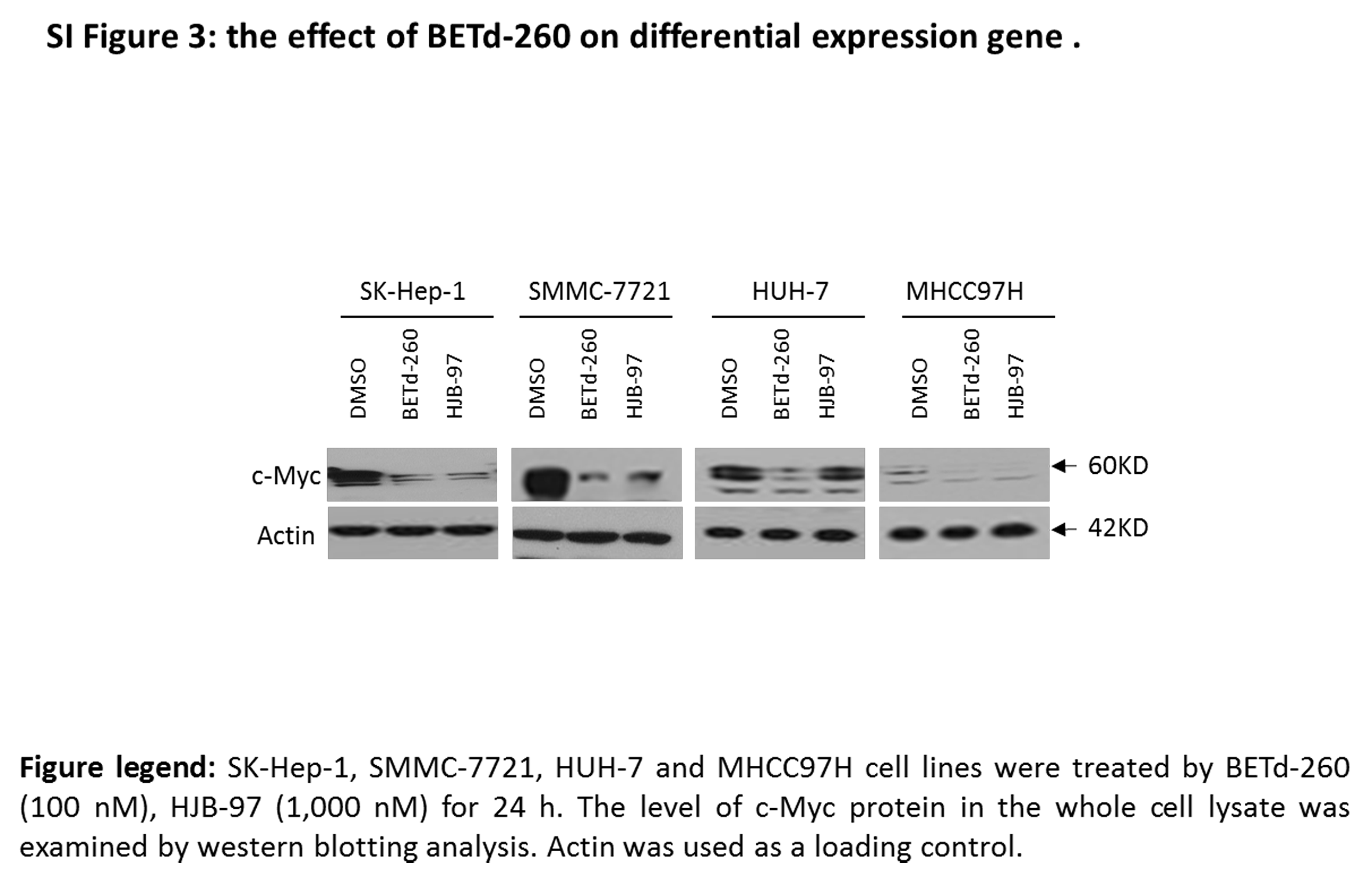

Supplement: Supplementary file 3 [file Image_3.TIF]
